# Quality of obstetric and newborn care in health centers of Addis Ababa City: using the WHO quality framework

**DOI:** 10.1186/s12913-023-09414-7

**Published:** 2023-05-09

**Authors:** Amaha Haile Abebe, Rose Mmusi-Phetoe

**Affiliations:** 1 Yeroam Consultancy, Addis Ababa, Ethiopia; 2 University of South Africa, Pretoria, South Africa

**Keywords:** Actionable information, Dignity, Effective communication, Effective referral. emotional support, Evidence-based routine care, Quality obstetric and newborn care

## Abstract

**Objective:**

The study aims to assess the quality of obstetric and newborn care using the WHO quality framework.

**Design:**

The study used explanatory sequential mixed methods design.

**Setting:**

This study was conducted in 50 health centers in Addis Ababa city administration from January 25 to December 31, 2021.

**Methods:**

A total of 50 health centers were surveyed using a structured questionnaire and 500 women in the postpartum period were interviewed using a structured questionnaire. Delivery records of the 500 women were reviewed using a structured checklist. A total of 338 midwives were interviewed using a structured questionnaire. The quantitative data was analyzed using Statistical Package for Social Sciences (SPSS).

**Results:**

The study revealed that only a third of the 50 health centers were providing good quality care (a quality score ≥ 75%). All the health centers had the physical resources (100%) to deliver obstetric and newborn care. The majority of the health centers had a system for actionable information (92%), functional referral (80%), and providing dignified care (80%). On the other hand, only a few of the health centers met the quality threshold for effective communication (24%), evidence-based practice of routine obstetric and newborn care (36%), and availability of mechanisms to support and motivate skilled birth attendants (24%). None of the health centers met the quality threshold for emotional support during labour and delivery. Obstetric caregivers’ high workload and job dissatisfaction were barriers to quality care.

**Conclusion:**

Ensuring quality obstetric and newborn care needs effective quality improvement interventions that aim to ensure women had effective communication, emotional support, and dignity during labour and delivery. Reducing the workload and increasing motivation of birth attendants play a critical role in improving the quality of care.

**Supplementary Information:**

The online version contains supplementary material available at 10.1186/s12913-023-09414-7.

## Introduction

Globally, the coverage of institutional deliveries has been significantly increasing over the past few decades. At the same time, a higher proportion of avoidable maternal and perinatal mortality and morbidity has also moved to health facilities, where poor quality care has become a challenge to the quest to end preventable mortality and morbidity [1].

The gap between countries with the highest and lowest mortality has increased despite the increased use of maternity care in high-mortality settings. This mismatch exposes an important gap in the quality of care. Poor quality care – delayed, inadequate, unnecessary, or even harmful services – minimizes the health gains for mothers and babies [2]. Similarly, evidence is growing that access to obstetric and newborn care services alone is not enough to improve newborns’ survival through the first day of life [3].

Improving the quality of existing obstetric and newborn care could have a significant impact on maternal and newborn mortality [4, 5]. Quality maternal health services that respond to local specificities require immediate attention to catalyze action and support the vision of global initiatives to achieve the SDG 3 global target of an MMR of less than 70 maternal deaths per 100 000 LB [2].

Moving beyond 2015, the WHO envisions a world where “every pregnant woman and newborn receives quality care throughout pregnancy, childbirth and the postnatal period” [1].

Governments should ensure that women have access to quality obstetric and newborn care, not only because it is a way to prevent maternal and newborn death but also because it is a fundamental human right that governments are obliged to fulfill [6, 7].

Defining quality of care is important for a common understanding of what it means and how to improve it. The 1965 work of Avedis Donabedian has been ground-breaking in understanding and measuring the quality of health care. Donabedian proposed using the triad of structure, process, and outcome to evaluate the quality of health care. He defined “structure” as the settings, qualifications of providers, and administrative systems through which care takes place; “process” as the components of care delivered; and “outcome” as recovery and survival [8].

**Fig. 1:**
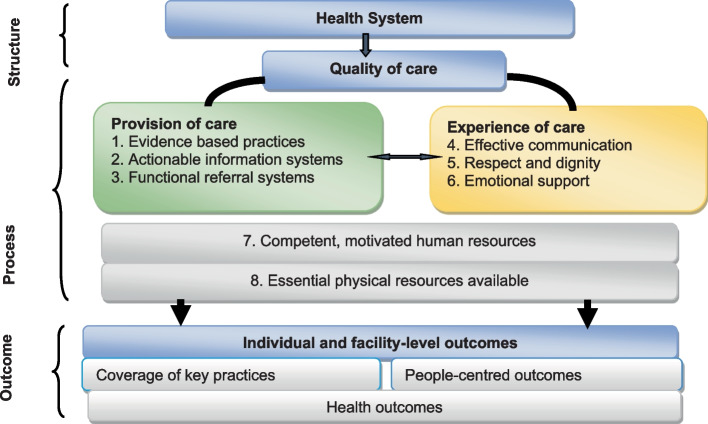
A WHO framework for quality of obstetric and newborn care [6]

Building on Donabedian’s health care quality model, the WHO developed a maternal and newborn health quality framework that conceptualizes QoC for maternal and newborn health (Fig. 1) [6].

The WHO quality framework is built on eight quality domains that transcend structure and process of care [6]. The eight quality domains are effective communication; emotional support; respect and dignity; evidence-based practice during pregnancy, childbirth and the post-partum period; availability of an actionable information system; effective referral; competent and motivated human resources; and physical resources [6].

This article is presented the quantitative part of a large mixed methods study that examined quality of obstetric care in Health Centers in Addis Ababa city using the WHO quality framework [6]. The study aims to develop a strategy to improve the quality of obstetric and newborn care.

## Research methods

### Study period and setting

The study took place from January 25 – December 31, 2021, in 50 health centers in Addis Ababa city, the capital of Ethiopia.

### Study design and methods

The study used an explanatory sequential mixed methods study design. A face-to-face structured interview with 500 women in the post-partum care quantitative phase was followed by an in-depth interview with 33 midwives and managers in the qualitative phase.

### Study population

The study population for the post-partum women survey was all women age 15–49 years old who had delivered babies and were attending immediate post-partum and post-natal care in health centers of Addis Ababa city during the study period and who fulfil the eligibility criteria. Exclusion criteria were women in the post-partum period who had delivered a baby at home or other health institution, women who had childbirth in the same health center but is in the first 6 h of delivery or after 6 weeks of delivery, who are very sick or have a sick new-born to take part in this study [9]. The study population and sampling methods for the postpartum women survey was published by authors in another article on respectful maternity care [9].

The study population for the midwives’ survey was all midwives aged 18 years and above who were working in maternity care at least for six months preceding the survey in the health centers selected for the study. The midwives who work in areas other than maternity care, those who had less than 6 months in the health center, and who are sick or suspected of COVID-19 were excluded from the study.

The study unit for the health centers survey was Health centers in Addis Ababa city administration that had been providing delivery care at least for the two years preceding the survey. This is because the outcome aspects of quality of care such as volume of service, institutional delivery, and perinatal and maternal death were measured for the Ethiopian Calendar Year 2012, a period covering at least 18 months before the survey. Health centers that never provided or did not provide delivery care for two or more years preceding the survey were excluded.

### Sample size and sampling technique

The sample size for the health centers’ survey was 50 health centers. There was a total of 97 health centers in Addis Ababa city administration of which only 90 health centers were providing delivery care during the study period and were eligible for the study. Fifty (55%) of the 90 health centers were included in the study. A stratified simple random sampling method was applied for the selection of health centers. Health centers were listed by sub-city and five health centers were selected from each of the ten sub-cities in Addis Ababa using a lottery method (Table 1).

**Table 1:** Sampling and sample size for quantitative and qualitative methods

Groups	Sampling method	Sample size	Assumptions
**Quantitative phase sampling and sample size**			
Survey of health centers	Sampling health center survey: A stratified simple random sampling method was applied for the selection of health centers. Health centers were stratified by sub-city. Lottery method was applied to select 5 health centers from each of the 10 sub-cities	**The sample size for the health centers survey (maternity unit heads interview, register review, and observation)** Health centers were stratified by the 10 sub-cities. Simple random sampling – a lottery method was used to select 5 health centers from each of the 10 sub-cities in Addis Ababa city	Half of the population was included in the study. A sample > 30 is sufficient to do a parametric test (Rule of thumb)
Survey of women in the post-partum period [9]	Sampling women survey: 10 women were interviewed from each of the 50 health centers Systematic random sampling was used to select 10 women who were 6 h to 6 weeks in the post-partum period. Women were selected from all eligible women who were in immediate post-partum care or who came to the health center for post-natal care during the 2 days visit to each of the health centers [9]	**The sample size for women interview** [9] ${n}_{1}={n}_{2}=\frac{{\left({z}_{a/2}\sqrt{2\overline{pq} }+{z}_{\beta }\sqrt{{p}_{1}{q}_{1}+{p}_{2}{q}_{2}}\right)}^{2}}{{\Delta }^{2}}$ $\mathrm{Calculate} \overline{p }=\frac{{p}_{1}+{p}_{2}}{2}.$ = 0.25 + 0.15 = 0.2 2 ∆ = p_1_-p_2_ = 0.25—0.15 = 0.1 n1 = n2 = (1.96 √.2*.8 + 0.84 √0.25*0.75 + 0.15*0.85)^2^ (0.1)^2^ n1 = 250 n2 = 250 n = n1 + n2 = 500 A study in Addis Ababa reported client satisfaction of 20% on intrapartum care [10]	P1 = % client satisfied with the care at facilities with high-quality score = 25% P2 = % client satisfied with the care at facilities with low-quality score = 15% 95% Confidence interval (Z ∝ /2 = 1.96) Power 80% (Zβ = 0.84)
Survey of midwives	Sampling midwives’ survey: All midwives working in the 50 health centers and available during the study period were interviewed using a structured questionnaire	**The sample size for midwives’ interview** *n* = (Z_∝/2_)^2^ pq d^2^ *n* = (1.96) ^2^ 0.53 (0.47) = 390 (0.05) ^2^ *P* = 0.53 q = 1-p = 0.47 d = 0.05 Correction for finite population nf = n/(1 + n/N) + 15% (n) addition for non-response nf = 390/(1 + 390/1200 + 15% (390) = 339 The sample size required after correction for a finite population was 339 midwives A study in Addis Ababa reported job satisfaction among midwives of 53% [11]	The proportion of midwives who were satisfied in their job = 53% The margin of error (d) = 0.05 95% Confidence interval (Z ∝ /2 = 1.96) N = 1200 number of all midwives in Addis Ababa

The sample size was 500 women in the post-partum period who had deliveries in the study health centers and attended immediate postpartum or post-natal care at these health centers. An equal quota of 10 women in the postpartum period was allocated for each of the 50 health centers selected for the study to ensure that all the health center quality scores are derived from an equal number of women interviewed per health center and are comparable across the study health centers. Systematic random sampling was used to select women attending immediate postpartum or post-natal care at the study health centers [9]. The study team had at least two days to visit each of the 50 health centers. Based on data from the health centers for the preceding two days, the total number of women in the postpartum period eligible for the study expected to visit the health centers in two study days ranged from 15 to 30 women [9]. The sampling fraction (K) was calculated by dividing the total number of women in the postpartum period expected to be served per health center in two days by the sample of women allocated to each health center, which is ten women per health center. The first woman was selected using a lottery method from 1 to K [9]. In facilities with fewer women, we interviewed every woman who fulfilled the eligibility criterion while for some facilities with many postpartum women, the sampling fraction (K) was two or three [9] (Table 1).

The total sample required for the midwives’ survey was 339 midwives. The total number of midwives in the fifty health centers is estimated to be 400 which is closer to the sample size estimated for the study. Therefore, all midwives who met the eligibility criteria and were available for the interview during the study visit at the 50 health centers were selected for the study (Table 1).

### Data collection instruments and operational definition

Women in the post-partum period, Midwives, and Health centers questionnaires were adapted from WHO obstetric and newborn care quality standard [6] and previous studies [12–14] on the topic. The questionnaires were tested for validity using face validity, content validity, criterion-related validity and construct validity [15]. The questionnaires were checked at face value if items measure the concept intended to measure. The concept, the conceptual framework, quality statements, and quality measures defined in the WHO quality standard for obstetric and newborn care [6] were the basis for the development of the study questionnaires, which better ensured that the concept was correctly measured through the survey questionnaires. A panel of experts (four midwives) was used to review the list of questions and response option relevance, clarity, and completeness to measure the concept of quality and respectfulness of obstetric and newborn care. Reliability is the "consistency" or "repeatability" of measures [15]. In this study, Cronbach’s alpha was used to test the internal consistency of items in the postpartum women’s survey questionnaire where the value was > 0.7 (0.938). The questionnaire was pre-tested in a similar setting to test its understandability, and completeness of response options.

Effective communication, emotional support, respect, and dignity during obstetric and newborn care were measured using data from interviews with the postpartum women. Evidence-based practice of obstetric and newborn care was measured based on data from a review of women’s delivery charts. Actionable information systems, effective referral, availability of human and physical resources, and quality improvement were determined based on data from the health center assessment questionnaire.

A composite index is a way of compiling one score from a variety of questions or statements that represent an attribute of a phenomenon that cannot be measured with a single question or statement [16]. Quality of care is a concept that cannot be measured with a single question or statement. Therefore, a composite index was constructed for quality of care, effective communication, emotional support, dignified care, evidence-based practice, actionable information, effective referral, and human and physical resources using an additive method that sums up several variables. The following composite indices were developed based on the WHO’s obstetric and newborn care quality framework and standards [6], a review of existing literature, and a panel of experts. A review of existing literature [12–14, 16] and the panel of experts (an obstetrician, two midwives, and a public health professional) were used to decide on setting 75% cut-off points to define and weight items that construct composite index variables.

Each of the composite indexes was constructed out of a set of statements or indicators that scored ‘0’ when the answer is no and ‘1’ when the answer is yes. The sum of scores was used to construct the score for each index variable and when the sum of scores was > 75% it is considered to meet the quality threshold. The following table summarizes how the index variables for the eight-quality domains were constructed (Table 2).

**Table 2:** The eight quality domains -Composite indices and indicators

Quality domains constructed based on variables from post-partum women interview and review of their delivery charts	Quality domains constructed based on variables from health centers’ survey
**Effective communication:** Six questions were used • Greetings at reception • Introduction by the provider • Communicated the way she understands • Provided adequate information • Provided the opportunity to ask questions • Informed consent Ten women were interviewed per health center. A health center was defined as delivering effective communication when the mean score from the 10 women is. ≥ 75% (≥ 4.5 out of 6 points)	**Actionable information system:** ten questions used • Clinical charts are available at all times for the routine recording of care • Standard forms and/or partograph used to monitor labour • There is a mechanism for information exchange among staff (handover meeting) • Has maternal and perinatal death reviews • Has a mechanism to implement findings of maternal and perinatal death reviews • Has HMIS /DHIS2 registers in place at all time • HMIS /DHIS2 registers are routinely administered • Has a data system for analysis and regular reporting • Management and staff meet periodically to review performance reports A health center was defined to have an actionable information system when it scored. ≥ 75% (≥ 7.5 out of 10 points)
**Emotional support:** Six questions were used • Encouraged to take fluid during labour • Encouraged to mobilize during labour • Encouraged to be labour position of her choice • Allowed to have a companion of choice • Companions are oriented to their role • Offered pain relief Ten women were interviewed per health center. A health center was defined as delivering emotional support when the mean score from the 10 women is. ≥ 75% (≥ 4.5 out of 6 points)	**Functional referral system:** eight questions used • Has a functional ambulance for referral • Has an up-to-date list of referral facilities • Has standard referral forms • Has reliable communication for referral and consultation (mobile or landline phone) • Has a formal agreement with referral centers • Registers are available for the referral of cases • regularly track and receive feedback on cases referred • always assigns professionals to accompany cases to referral facilities A health center was defined to have a functional referral system when it scored. ≥ 75% (≥ 6 out of 8 points)
**Dignified Care:** Six questions were used • Clean delivery area • Delivery area ensures privacy • Delivery care ensures s confidentiality • Women did not have verbal abuse • Women did not have physical abuse • Women were not neglected • Ten women were interviewed per health center. A health center was defined as delivering dignified care when the mean score from the 10 women is. ≥ 75% (≥ 4.5 out of 6 points)	**Availability of mechanism to support and motivate staff:** eight questions used • Has standard procedures for recruitment & deployment of all staff • Has plans for recruitment & deployment of staff • Has periodic appraisal of the performance of staff • Has performed staff performance appraisal in the past year • Has a list of staff on rotation /duty posted in the labour room • Has the address and telephone number of staff posted for consultation and communication • Has a formal agreement with referral centers • Most staff had training or refresher on BEMONC • Most staff have received their job description • Most staff oriented on their job description A health center was defined to have a functional mechanism to motivate and support staff when it scored. ≥ 75% (≥ 7.5 out of 10 points)
**Evidence-based routine obstetric and newborn care:** was measured using 34 questions • 6 questions were used to assess essential obstetric history at admission’ • 6 questions were used to assess essential physical examination at admission • 11 questions were used to measure follow and management of labour • 6 questions on newborn care • 5 questions were used to assess the practice of basic laboratory tests during labour Ten women’s delivery charts were reviewed per health center. A health center was defined as delivering Evidence-based routine obstetric and newborn care when the mean score from the 34 women is. ≥ 75% (≥ 25.5 out of 6 points)	**Availability of physical resources: 58** questions or indicators used • 14 indicators for the availability of infrastructure and facility (rooms, coach, electric, water) • 20 indicators for the availability of essential obstetric and new-born care equipment • 24 indicators for the availability of essential drugs and supplies A Health center was defined to have the physical resources to deliver obstetric and newborn care when it scored. ≥ 75% (≥ 43.5 out of 58 points)

#### Midwives’ job satisfaction

Midwives’ job satisfaction was assessed using 20 job-related parameters. Each of the 20 items was scored out of five points: Very dissatisfied = 1, dissatisfied = 2, not sure = 3, satisfied = 4, and very satisfied = 5. The 20 indicators were added to construct a composite index for midwives’ job satisfaction. The 20 items were scored out of a total of 100 points. A midwife was defined as satisfied with the job when he or she scored at least 75%.

A mean job satisfaction score was calculated from the total number of midwives interviewed per health center. A health center was defined as having midwives satisfied with their jobs when the health centre’s mean job satisfaction score was at least 75%.

#### The workload of birth attendants

The workload of the skilled birth attendants (SBAs) working in maternity care was calculated by dividing the annual number of deliveries attended per health center to the total number of SBAs working in maternity care.

#### Quality of obstetric and newborn care

The quality of obstetric and newborn care was assessed using eight quality domains with a total of 138 indicators that included effective communication (6 indicators), emotional support (6 indicators), dignified care (6 indicators), the practice of evidence-based routine obstetric and newborn care (34 indicators), actionable information system (10 indicators), functional referral system (8 indicators), availability of a mechanism for competent and motivated human resources (10 indicators), availability of physical resources (58 indicators).

Quality of care (QoC) was defined based on equally weighted scores for the above eight quality domains. The health center scores for each of the above eight quality domains were converted to percentages. A quality score which is a mean score for the eight domains was calculated out of 100%.

A health center was defined as having good quality obstetric and newborn care when the health center’s mean quality score was 75% and above. A health center was defined as having medium-quality obstetric and newborn care when the quality score was 50% to 74.99%. A health center was defined as having poor quality obstetric and newborn care when the quality score was below 50%. The cut-off point to define the quality of obstetric and newborn care was set based on the opinion of a panel of experts and a review of a similar study [17].

### Data collection

Structured face-to-face interview with 500 women in the post-partum period and 338 midwives was conducted by four experienced midwives trained for 5 days. Women and midwives who fulfilled the eligibility criteria were provided information about the study’s aim, risks, benefits and their rights not to participate or terminate the interview at any time. Respondents who agreed to participate signed written consent. Respondents were interviewed face to face. Each respondent was asked each question on the questionnaires as it reads on the question and recorded responses on the questionnaires. Informed consent was obtained from the women in post postpartum period to review their delivery records. Their delivery records were retrieved and reviewed using a structured checklist.

The health center survey questionnaire was administered through interviews with maternity unit heads, observation of facilities, infrastructure, equipment, drugs, and supplies, and a review of registers. The head of the maternity unit responded to the questions of the health center survey questionnaire and assisted the research assistants to administer the survey questionnaire through observation of facilities, infrastructure, equipment, drugs, and supplies and a review of registers and records.

### Data analysis

The data from the three questionnaires that have been cleaned were entered into Epi data by a data encoder. Then it was exported from Epi-data to Statistical Package for social sciences (SPSS) version 20 for data coding and analysis. The data from the women midwives and health centers survey was coded, and index variables were constructed. Once the three data sets for postpartum women, midwives, and health center surveys were coded, recoded and index variables constructed, a fourth database was created combining selected variables and index variables from the women in the postpartum period and midwives survey data to the health center survey SPSS data. Mean scores were calculated per health center for women in the postpartum period and midwives’ survey variables and index variables were selected to be merged into the health center data. Then the mean score of a health center for women in the postpartum period and midwives interviewed from each health center was entered into the health center database to create the fourth database which combined the health center data with selected variables from the women in the postpartum period and midwives’ survey data. Then the next step was to run descriptive statistics that include frequency (percent or proportion), mean, median, mode, and standard deviation. Multiple linear regression was used to test the relationship between the dependent (quality of obstetric and newborn care) and the independent variables.

### Ethical considerations

Ethical clearance was obtained from the ethics committee of the University of South Africa (UNISA) prior to conducting the study. The consent to participate in the study was voluntary. Informed consent was obtained from all subjects and/or their legal guardian(s). All methods were carried out in accordance with relevant guidelines and regulations. All other universal ethical principles relating to research with human subjects were observed.

## Results

### Profile of respondents

All the 50 health centers selected for the study were surveyed with an interview of maternity unit heads, observation, and review of registers. All 50 health centers were providing labour delivery service was provided 24 h a day and seven days a week. Only 24% (*N* = 12) of the health centers were providing C-section delivery during the study period. A third of the health centers had a catchment population of more than 40,000 which is above the ministry of health standard (Table 3).

**Table 3:** Health Centers General Information (*N* = 50)

Sociodemograpic Variables	Frequency	Percent
Population served		
Urban	25	50
Both Urban and rural	25	50
Does the health facility MNCH service supported by any non-governmental organization?		
No	39	78
Yes	11	22
Does the facility provide Caesarian delivery		
No	38	76
Yes	12	24
Catchment population served per health center		
≤ 40,000 People	32	64
> 40,000 People	10	36
Number of skilled birth attendants providing maternity care per health center		
≤ 10	13	26
11–15	15	30
16–20	12	24
≥ 21	10	20
Number of deliveries attended per year per skilled birth attendant		
1–50 deliveries per SBA per year	20	40
51–100 deliveries per SBA per year	20	40
> 100 deliveries per SBA per year	10	20

The majority (72%) of women were in the age group of 20 to 29 years. The mean and median age of women was 26.5 and 26 years respectively (Standard deviation of 4.5 years). The majority, 89%, of women were currently married. The majority (96%) of women had formal mostly elementary or high school education. Most (71%) of women were unemployed.

A total of 338 midwives were interviewed using structured questionnaires. The majority, 74%, of midwives in the study were female. Most, 61.8%, midwives were in the age group 25 to 29 years the rest 23.7% were in the age group 30 to 34, 5.9%, were younger than 25 years and 8.6% were older than 35 years. 58.9% had a bachelor’s degree and 39.9% had diplomas. Only 1.2% of the midwives had a master’s degree. Most 67.7% of the midwives had four to nine years of work experience. Midwives who had one to three years of work experience constituted 23.1% of the sample and 9.2% of midwives had ten or more years of work experience.

### Quality of Obstetric and newborn care

Only 34% (*N* = 17) of the health centers were providing good quality obstetric and newborn care (a quality score ≥ 75%). The rest, 66% (*N* = 33), of the health centers were providing medium-quality obstetric and newborn care (a quality score of 50.01%-74.9%). None of the health centers fell in the poor-quality care category – a quality score below 50%. The mean score for quality of obstetric and newborn care was 73% with a SD of 6. The minimum quality score of health centers was 57% and the maximum score was 88%.

**Fig. 2:**
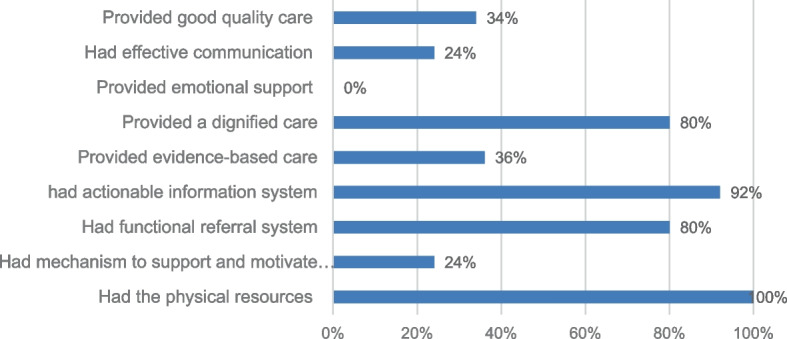
Percentage distribution of health centers that met the good quality threshold for the eight obstetric and newborn care quality domains (*N* = 50)

The majority, 80% (*N* = 40), of the health centers offered dignified care (mean score ≥ 75%). However, only 24% (*N* = 12) of the health centers met the quality threshold for effective communication (mean score ≥ 75%) and none of the health centers met a 75% mean score threshold for emotional support during childbirth. Only 36% (*N* = 18) of the health centers were providing evidence-based routine obstetric and newborn care – a mean score ≥ 75% (Fig. 2).

All the health centers (*N* = 50) had the physical resources to provide quality obstetric and newborn care at their level (Score ≥ 75%). The majority, 92% (*N* = 46), of the health centers had actionable information systems (score ≥ 75%). There were effective referral systems in 80% (*N* = 40) of the health centers (score ≥ 75%). However, only 24% (*N* = 12) of the health centers had a mechanism to support and motivate staff—a score ≥ 75% (Fig. 2).

The mean score for quality care in the health centers was 73%. Of the eight quality domains, the highest mean score for quality was for availability of physical resources recording 92%, followed by the availability of an actionable information system with a mean score of 89%, and dignified care with a mean score of 85%. The fourth in rank was the availability of a functional referral system with a mean score of 81% followed by evidence-based practice of routine obstetric and newborn care with a mean score of 72%. The three quality domains with the lowest quality score were availability of a mechanism to support and motivate staff, effective communication, and emotional support, with mean scores of 64%, 60%, and 42% respectively. Emotional support during childbirth was the least performed quality domain (Table 4).

**Table 4:** Distribution of health centers’ mean percentage score for the eight obstetric and newborn care quality domains (*N* = 50)

Quality of care domains	Mean	Median	Std. Deviation	Minimum	Maximum
Experience of effective communication	60	60	18	12	88
Experience of emotional support	42	38	13	18	73
Experience of dignified care	85	87	10	62	100
The practice of evidence-based routine obstetric and newborn care	72	71	11	51	95
Actionable information system	89	90	10	60	100
Functional referral system	81	81	15	38	100
Mechanism to motivate staff	64	65	15	30	90
Availability of physical resources	92	93	5	78	100
The overall quality of care score	73	73	6	57	88

### Factors influencing the quality of obstetric and newborn care

Multiple linear regression was used to assess the association between the dependent variable (health centers’ quality score) with the independent variables. The independent variables include support from a non-governmental organization, availability of QI mechanism, availability of C-section delivery, midwives’ workload, and job satisfaction.

The workload and job satisfaction of the midwives were the only factors that had statistically significant associations with the quality of obstetric and newborn care. Health centers’ obstetric and newborn care quality scores had a statistically significant association with birth attendants’ workload and job satisfaction. An increasing number of deliveries per birth attendant per year (workload) had a negative effect on the health centers’ quality score (*P* = 0.012). On the other hand, health centers with higher midwives’ job satisfaction scores had higher QoC scores (*P* = 0.042).

Quality of obstetric and newborn care had no statistically significant association with the availability of QI mechanism, availability of C-section delivery, and whether the health center had support from non-governmental organizations (*P* > 0.05) Table 5.

**Table 5:** Predictors of health center quality of care score (*N* = 50)

Independent variables (predictors)	Unstandardized Coefficients		Standardized Coefficients	t	Sig	95.0% Confidence Interval for B	
	B	Std. Error	Beta			Lower Bound	Upper Bound
(Constant)	58.557	8.418		6.96	.000	41.622	75.492
Workload (number of deliveries per midwife)	-.038	.014	-.342	-2.62	.012	-.067	-.009
Mean midwives’ job satisfaction’ score out of 100 points	.243	.116	.273	2.09	.042	.009	.476

## Discussion

Ensuring access to obstetric and newborn care alone is not enough to meet the SDG goal it needs to ensure women receive quality obstetric and newborn care once reached at the health facilities [1, 2]. However, the quality of obstetric and newborn care was largely sub-optimal. In this study, only 34% of the health centers were providing good quality obstetric and newborn care (a quality score ≥ 75%). Similarly, A study in Bangladesh among 852 health facilities also reported that only 33% of health facilities were providing good quality obstetric care – a quality score of 75% or more [18]. A study in India among private health facilities also reported that three fourth of the health facilities were providing poor quality obstetric and newborn care [19].

In this study, Health centers were performing well on the availability of physical resources, an actionable information system, a functional referral system, and providing dignified care. However, most health centers performed poorly in effective communication, emotional support, evidence-based practice of routine obstetric and newborn care, and availability of mechanisms to support and motivate staff. Studies in different parts of the world also reported that facilities are mostly doing better in availing the physical resources and systems such as referrals and information. However, there is mostly a gap in practice that includes communication, emotional support, and evidence-based practice [20-22]. Lack of motivated birth attendants 24 h a day and 7 days a week was also reported as one of the quality breaches in other studies [18, 19].

Midwives’ volume of work and job satisfaction were the factors that had statistically significant associations with the quality of obstetric and newborn care. Health centers’ QoC score has a statistically significant association with birth attendants’ volume of work (number of deliveries per SBA per year) and job satisfaction. Increasing numbers of deliveries per birth attendant per year (volume of work) had a negative effect on health centers’ quality score (*P* = 0.012). Health centers with higher midwives’ job satisfaction scores had higher QoC scores (*P* = 0.042). Similarly, Dieleman and Harnmeijer noted that providers satisfied with their jobs were more likely to be motivated and perform better in all aspects of care including providing respectful care and communication with clients, adhering to evidence-based guidelines and documentation of care [23].

The strength of the study lies in the consistent application of the WHO quality framework that comprehensively illustrates the quality of obstetric and newborn care not only from the perspective of provision of care but also from the dimension of how women experience this care. In addition, the study collected data from clients, and service providers and observed the health facilities for the availability of resources. The weakness of this study was the fact that the study used interviews about women’s experience of childbirth and review of patient delivery records instead of direct observation of obstetric and newborn care which minimized the objectivity of measurement of the quality of obstetric and newborn care. In addition, this study did not assess all 318 quality measures defined under the 31 quality statements of the WHO quality standard [6].

## Conclusion

Birth attendants’ availability and job satisfaction were the key determinants of the quality of obstetric and newborn care in health centers of Addis Ababa city. Therefore, Addis Ababa City Health Bureau needs to introduce an effective mechanism for the motivation of birth attendants and ensure that there are an adequate number of birth attendants commensurate to the volume of work in each health center.

Effective communication and emotional support during childbirth are domains of quality least performed in health centers of Addis Ababa city. It needs to train providers and develop policies and guidelines to ensure providers adhere to standards of respectful maternity care particularly communication, emotional support, and dignity. The health centers need to adopt the new WHO obstetric and newborn care evidence-based guidelines for labour [24] and train providers to the evidence-based practice guidelines in follow-up and management of labour and newborn care.

Ensuring the quality of obstetric and newborn care in health centers of Addis Ababa city needs establishing and running an effective quality improvement mechanism that includes quality units, trained personnel, tools and a budget to plan, assess and improve quality on regular bases.

The WHO quality framework [6] provides a comprehensive quality standard and indicators to assess and improve the quality of obstetric and newborn care. However, the WHO quality framework has too many indicators (318 indicators) and lacks prioritization of indicators. It does not provide enough guidance on how to measure and score the list of indicators. In addition, the WHO quality framework does not have a tool and there is no unified global tool that can capture the comprehensive list of indicators for maternal and newborn health set forth by the WHO quality framework and standards [25].

Therefore, the tools and methods used in this study to measure the list of obstetric and newborn care quality indicators defined in the WHO quality framework can serve as a basis to develop and implement obstetric and newborn care quality assessment and quality improvement in health centers of Addis Ababa City Administration.

## Supplementary Information

**Additional file 1: S table 1.** Percentage distribution of women in the postpartum period by sociodemographic characterstics (*N*=500)^9^. **S table 2**. Midwives sociodemograpich characterstics (*N*=338).

## Data Availability

The datasets used and/or analyzed during the current study are available from the corresponding author on reasonable request.

## Abbreviations

SBA: Skilled birth attendant
QoC: Quality of care
UNISA: University of South Africa
WHO: World Health Organization
