# Supplementary material for: Quality of obstetric and newborn care in health centers of Addis Ababa City: using the WHO quality framework
Source: BMC Health Serv Res. 2023 May 9;23:459. doi: 10.1186/s12913-023-09414-7 (PMC10169211; doi:10.1186/s12913-023-09414-7)
Supplement: Supplementary file 1 — Additional file 1: S table 1. Percentage distribution of women in the postpartum period by sociodemographic characterstics (N=500)9. S table 2. Midwives sociodemograpich characterstics (N=338). [file 12913_2023_9414_MOESM1_ESM.docx]

Supplemental tables

S table 1: Percentage distribution of women in the postpartum period by sociodemographic characterstics (N=500)^9^

| Sociodemograpic Variables | Frequency | Percent |
| --- | --- | --- |
| Age  15-19 year  20-24 year  25-29 year  30-34 year  >/=35 Year | 21  164  193  77  45 | 4.2  32.8  38.6  15.4  9.0 |
| Marital Status  Never married  Married/living together  Divorced/separated/Widowed | 47  447  6 | 9.4  89.4  1.2 |
| Educational Status  No Formal education  Primary (grade 1-8)  Secondary (grade 9-12)  College education and above | 44  238  138  80 | 8.8  47.6  27.6  16.0 |
| Employment status  Employed in Govt, NGO or Private organization  Self Employed  Not Employed | 68  76  356 | 13.6  15.2  71.2 |
| Family Monthly Income based on tax category  ≤1650 birr (≤33.00 USD)  1651-3200 birr (33.01-64.00 USD)  3201-5250 birr (64.01-105.00 USD)  ≥5251 birr (≥105.01 USD)  Not Reported/disclosed | 73  192  117  91  27 | 14.6  38.4  23.4  18.2  5.4 |
| Place of residence  Within Addis Ababa City  Outside of Addis Ababa city | 462  38 | 92.4  7.6 |
| Number of children alive  one  Two  Three  Four or More | 218  167  77  38 | 43.6  33.4  15.4  7.6 |

S table 2: Midwives sociodemograpich characterstics (N=338)

| Sociodemograpic Variables | Frequency | Percent |
| --- | --- | --- |
| Sex  Male  Female | 88  250 | 26  74 |
| Age  20-24 year  25-29 year  30-34 year  >/=35 Year | 20  209  80  29 | 6  62  24  9 |
| Years of work experience  1-3 Years  4-6 Years  7-9 Years  ≥ 10 Years | 78  139  90  31 | 23  41  27  9 |
| Work experience in the current health center  1-3 Years  4-6 Years  7-9 Years  ≥ 10 Years | 104  141  76  17 | 31  42  22  5 |
| Educational status  Diploma  Bachelor’s degree  Master’s degree and above | 135  199  4 | 40  59  1 |
